# Periprosthetic Fracture of Greater Trochanter in Total Hip Replacement Stemming from Pin Site Placement in Navigation-Assisted Surgery

**DOI:** 10.1155/2019/1945895

**Published:** 2019-04-04

**Authors:** Ava Brozovich, David R. Lionberger

**Affiliations:** ^1^Houston Methodist Hospital, Houston, TX 77030, USA; ^2^Texas A&M College of Medicine, Bryan, TX 77807, USA

## Abstract

Surgeons are looking to use computer computer-assisted surgery (CAS) in total hip arthroplasty (THA) in order to quantify leg length measurement, angular cup placement, and enhance stability to provide enhanced accuracy in implant placement. As a result, CAS in THA is gaining popularity. This technology employs the use of pins and provides the surgeon with real-time feedback on positioning intraoperatively. Previous total knee arthroplasty (TKA) literature has reported pin-associated complications such as infections, neuropraxia, and suture abscess. To our knowledge, there have been reports of tibial stress fracture after CAS TKA, but this is the first report of a pin causing fracture of the greater trochanter leading to dislocation in THA. Further studies may be warranted to optimize pin placement for trackers to prevent fractures of the greater trochanter.

## 1. Introduction

Total hip arthroplasties (THA) are routinely performed and have a positive impact on patients' lives [1–3]. In addition, calculations have estimated that by 2030, the demand for primary THA will grow by 174%, with orthopedic surgeons performing 572,000 annually [4, 5]. However, despite impressive patient satisfaction after surgery, orthopedic surgeons still struggle to attain joint stability and leg length equality and reduce wear and/or dislocation [6, 7]. Even more importantly, surgeons are aware of potential legal implications for component position inaccuracy as these complications represent the bulk of legal cases following THA. Many studies have demonstrated that surgeries using computer-assisted surgery (CAS) technologies have statistically improved accuracy to ensure leg length, cup position, and stability [8, 9].

However, as with any new technology, there is a risk of potential complications. The CAS camera tracker requires several pins to be placed in the iliac crest and greater trochanter of the femur. There are several reports of complications associated with the pelvic pins; however, there are no known reports related to pins placed in the femur [10, 11].

We report this complication of CAS not to discourage the use of navigation but instead alert surgeons to potential risks when using CAS. In addition, this case report serves as a reminder to be careful in the placement of navigation pins to prevent occurrence of fractures.

Patient consent was obtained for this case report. The authors declare that there is no conflict of interest regarding the publication of this paper.

## 2. Case History

**Figure 1:**
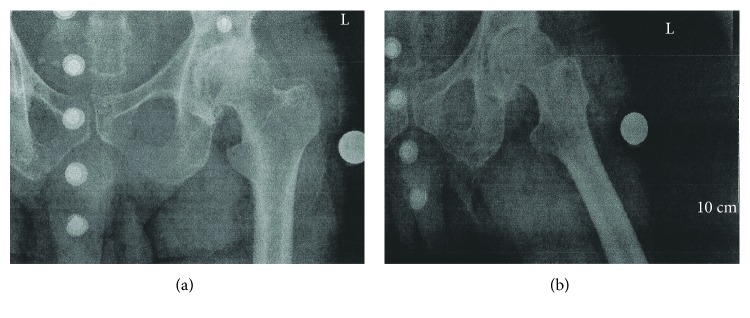
Preoperative AP and lateral X-rays showing stage IV osteoarthritis of the left hip.

The patient is a healthy 66-year-old male that presented with a five-year history of restricted motion and pain of the left hip associated with Kellgren-Lawrence stage IV arthritis (Figure 1).

**Figure 2:**
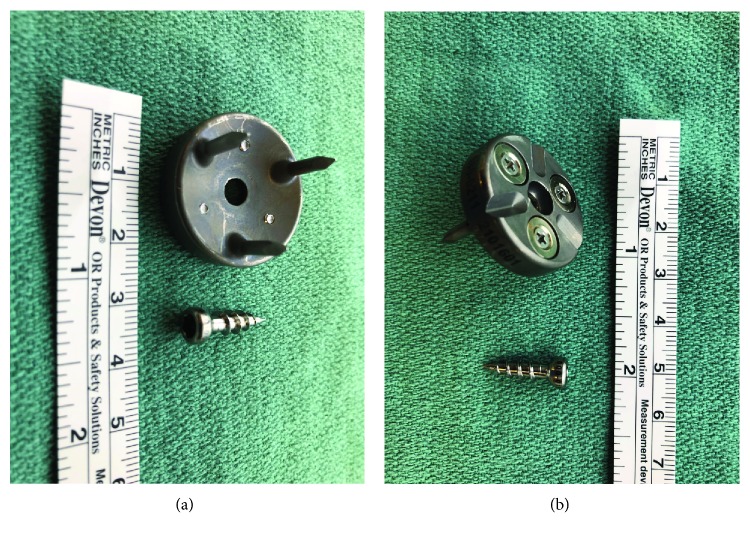
Tracker mount and pins utilized in CAS.

As a result, the patient had a left THA utilizing a posterior approach and CAS under general anesthesia. Navigation for the total hip was utilized to optimize radiographic alignment position offset and lengthening parameters. This was done by first acquiring predetermined landmarks to determine the center of the joint after stabilization of the camera in the anterior superior crest with two pins. Once the femoral head center was established, the CAS was digitized to acquire the femoral shaft to triangulate the pelvis, acetabulum hip center, and femur. While the femoral tracker was placed for the best camera visualization, effort was also taken to place the tracker on the flat lateral side of the trochanter. The CAS camera and tracker placement consists of a camera mounted on the superior crest of the ileum. The femoral tracker is fixed to the lateral greater trochanter. Usual placement is three cm below the tip of the greater trochanter on the posterior lateral border.  Figure 2 displays the pins and femoral tracker base that the camera is placed on.

Of note, intraoperatively additional retraction around the tracker mount on the femoral side of the joint at the level of the iliotibial band was needed to prevent tracker interference during rotation of the hip joint to measure alignment. Because the fascia is strong enough that it can alter the tracking accuracy, it was necessary to utilize retraction, which may have added additional stress on the tracker base.

**Figure 3:**
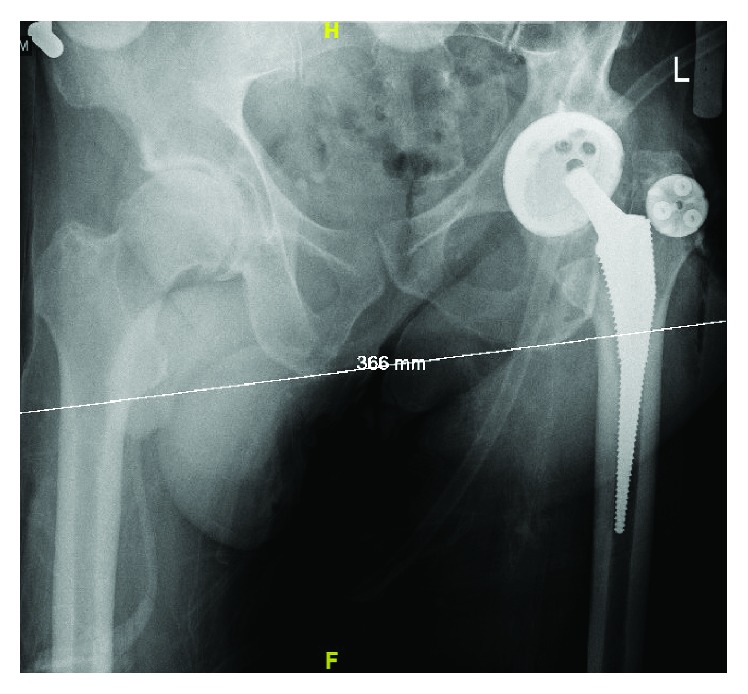
Intraoperative X-ray of the left hip demonstrating satisfactory positioning of the implants. The tracker base for the camera is visualized on the greater trochanter of the femur.

An intraoperative pelvis X-ray (Figure 3) was taken as a record to ensure accuracy, because CAS is a new technology. Intraoperative X-ray confirmed appropriate placement of the implant and was consistent with computer values of 43 degrees abduction and 20 degrees anteversion; offset was 0 and leg length was +2 mm. The target acetabular placement was 35-50 degrees abduction and 15-20 degrees anteversion. There were no intraoperative complications or any obvious evidence of compromise to the greater trochanter or the canal when a trial reduction was performed. In addition, the leg was taken through a full range of motion and demonstrated excellent stability of the hip joint. A Zimmer 58 G7 cup, G 40 mm liner, 40 3+ ceramic head, and 7 mm Taperloc complete high offset stem were utilized in the operation. The patient tolerated the procedure well and was sent to the recovery room in stable condition.

**Figure 4:**
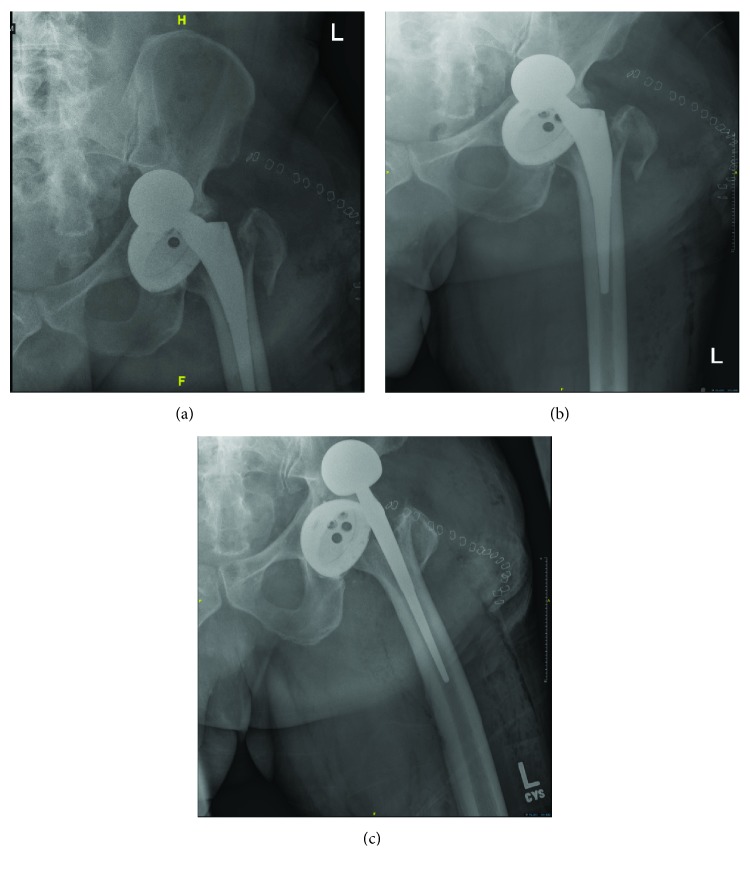
AP and lateral X-ray of the left hip 1 postoperative day after insertion of implants demonstrating anterior dislocation of left femoral head prosthesis and fracture of the greater trochanter.

Postoperatively, the patient received apixaban (Eliquis), hydrocodone-acetaminophen for pain control, and bilateral sequential compression devices. The patient remained restricted to the bed and placed on fall precautions until occupational therapy visited the patient on postoperative day one. The patient did not report a fall, but did note a “grinding sensation” on weight bearing. Physical exam revealed shortening of the left lower extremity. An X-ray demonstrated an anterior dislocation of the left femoral head prosthesis and fracture of the greater trochanter (Figure 4). Consequently, the patient was taken back to the operating room one day after THA for an open reduction of the dislocation with internal fixation of the trochanteric fracture. Intraoperatively, it was determined that there was an anterior dislocation of the hip and a greater trochanteric fracture, close to the placement of the tracker pins. There did not appear to be instability or damage to the lesser trochanter. The integrity of the fixation of the femoral component and acetabular cup was intact and not damaged. The cup version was not changed from the original position.

**Figure 5:**
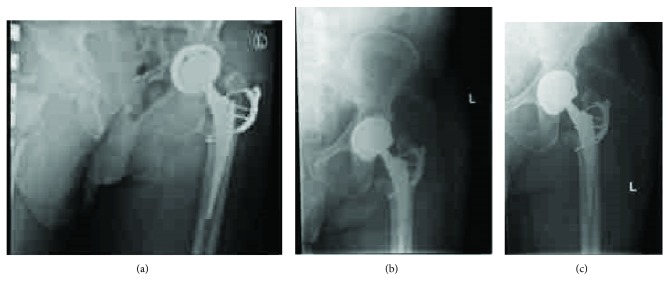
Pelvic X-ray of the left hip one postoperative day of THA revision demonstrating satisfactory positioning of the implants and placement of trochanteric cable plate. Wire was placed around the inferior portion of the Zimmer 51 mm short stature plate at the lesser trochanter and below. A second wire was placed at the upper trochanter at the implant junction. A third wire was placed around the greater trochanter.

**Figure 6:**
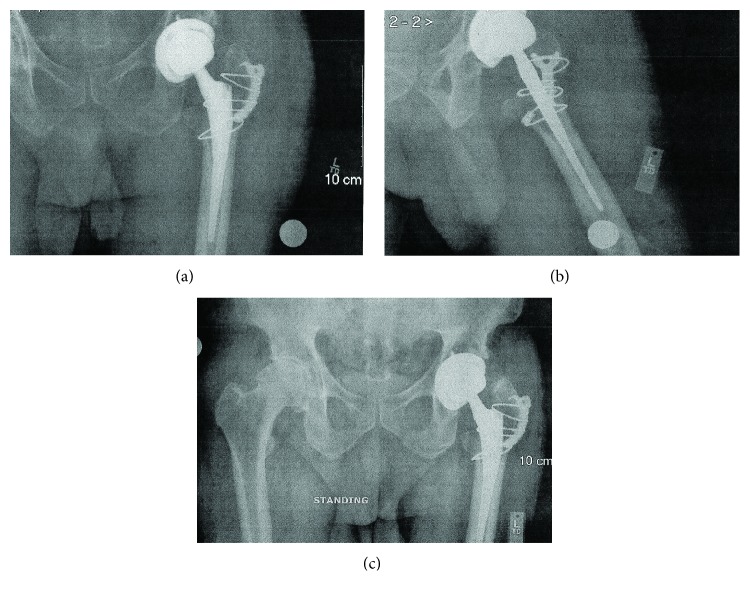
AP and lateral hip and standing pelvic X-ray at a six-week postoperative visit that demonstrate satisfactory positioning of the implants and trochanteric cable plate.

Postoperatively, X-ray findings confirmed correct alignment (Figure 5). Postoperatively, the patient was placed an anterior hip dislocation precautions, which included refraining from leaning backwards, rotating the knee outward, sudden forceful movements, and avoiding stress on the incision site. At a six-week postop, the patient began full weight bearing and did not complain of any lateral trochanteric pain. A six-week postoperative X-ray demonstrated (Figure 6) optimal position of the plate without evidence of mechanical failure, breakage, or erosive changes. Two view hip series demonstrated proper alignment of the implant, fracture healing without callus formation, and a reduced fracture. Range of motion of the hip was approximately 90% of the maximum rotation and flexion. Gross evaluation of the hip was normal with a well-healed incision without any evidence of an infectious process.

## 3. Discussion

Computer-assisted navigation technology is utilized intraoperatively to provide surgeons real-time feedback on the position and orientation of implants [12], allowing for less variation in implant position [13–18]. The use of pins is a necessary part of computer navigation and has been associated with complications such as infections and transient neuropraxia [10, 19–21]. It is hypothesized that the pin site complications could be directly associated with the necrosis caused from the drilling through or near the cortical bone to place the pins [22].

This is the first reported case of periprosthetic fracture in THA that is associated with the pin sites. Conversely, in TKA, there are many previous studies that have demonstrated tibia fractures (0-15%) postoperatively [23–25]. Though prior studies on THA have not reported any periprosthetic fractures, some have noted complications such as infection, neuropraxia, and suture abscess [10, 11]. Though no fractures have been reported following THA, biomechanical studies have demonstrated that there is a 40-70% decrease in bone strength of after creating a drill hole for placement of the tracker. Therefore, the pin holes may act as a biomechanical stress riser and it is hypothesized that the decrease in bone strength is due to the creation of screw holes and transcortical drilling [26].

There is little information on when the bone is most at risk for infection or fracture after the removal of the screw, and experts are still debating in what time frame after pin placement the bone is most at risk for fracture [27]. This case occurred within 1 postoperative day of pin placement, and though it was most likely due to the pin placement, it would have also been due to an undetected crack or saw injury during the cut of the femoral neck intraoperatively.

Moreover, the construct of the base of the greater trochanteric tracker disc (2.4 cm by 7 cm) utilized in this case poses potential additional complications. The base mount includes 4 3/32 inch diameter pins in variable lengths from 1/4 to 3/8 inch. The tracker base is the affixed with a single cancellous screw, which creates an additional stress riser (Figure 2). Though each isolated component does not create significant stress, a summation of all components at the level of the cortical bone under tension creates a stress riser [26]. When examining subsequent cases to determine a cause other than the abovementioned stress riser, it is conceivable that added traction of the proximal femur to provide visualization to the tracker may cause excessive stress to the trochanter. When not using CAS, this added tissue retraction is not necessary. Therefore, the process of using CAS, in addition to the hardware of CAS on the trochanter, may be the tipping point for fracture propagation.

Furthermore, in this case, it is most likely that the fracture of the greater trochanter led to the anterior dislocation of the femoral head prosthesis. Intraoperatively, it was determined that the acetabular component was aligned. CAS values for the cup and femur were 43 abduction and 20 degrees anteversion, respectively. Due to the alignment obtained intraoperatively, the components of the revision were not changed from the original surgery. Lewinnek et al. previously reported that dislocation of the hip if the acetabular was in 5-25 degrees of anteversion and 30-50 degrees of abduction was four times less likely than the CAS measurements in this reported case [28]. As the patient was in the “safe zone” intraoperatively, it is likely the absence of trochanteric stabilizing forces is the principle contribution to the dislocation in this case.

Limitations of this case report include that it is unknown if the fracture occurred first or the hip dislocation of the hip occurred first. However, if we assume normal position and hip dynamics, it can be presumed that if the fracture had not occurred, the dislocation would have been prevented. In addition, the patient had no significant trauma postoperatively, and even if the patient did dislocate their hip due to poor component position, the periprosthetic fracture should not have occurred. Furthermore, the stresses due to the navigation pins are strong enough to cause the fracture of the femur, because of the stress riser from the pin site placement. Therefore, this report suggests that the tracker should possibly be placed more posteriorly, where there are less tensile forces from abductors in order to avoid fractures of the femur. Moreover, additional research and development may be needed to determine the placement of pins to allow CAS to have utility, but decrease the amount of biomechanical stress on the bone. This may include development of an alternative strategy to mount the camera and tracker without the use of pins to avoid additional stress to the femur or injecting a gel or glue into pinholes following the removal of the pins to prevent the propagation of potential fractures.

## 4. Summary

As computer-assisted navigation is more commonly used by surgeons, complications such as fractures may be a risk. There is also a potential need to adopt less invasive tracker mounts, which do not damage the cortical strength of the femur. In addition, surgeons should be vigilant in retraction of the femur especially around the tip of the trochanter by placing force distal to the tracker base. While, it may be beneficial to mount the tracker base at a different position to prevent injury to the cortical bone, design changes of the tracker base may also be necessary. However, it is hoped that by alerting surgeons to these potential complications, such problems will be minimal in the future.
